# The effect of emotion regulation difficulties and loneliness on anxiety, depression, and stress levels in remote workers

**DOI:** 10.1186/s12889-025-23855-1

**Published:** 2025-07-29

**Authors:** Ulaş Korkmaz, Meltem Hazel Şimşek, Ömer Faruk Şahin

**Affiliations:** 1 Department of Psychiatry, Giresun University, Giresun, Türkiye Turkey; 2 Faculty of Medicine, Giresun University, Giresun, Türkiye Turkey

**Keywords:** Remote work, Emotion regulation, Loneliness, Mental health

## Abstract

**Background:**

While remote work brings flexibility to work life, it can also bring loneliness, emotion regulation difficulties, and some mental health symptoms. This study examined the relationship between loneliness and emotion regulation difficulties and levels of depression, anxiety, and stress in remote workers and the role of some sociodemographic variables in these relationships.

**Methods:**

An analytical cross-sectional observational study was conducted. One hundred twenty-one participants (53.7% female, 46.3% male), aged 23–56 and working remotely for at least six months, were reached through snowball sampling and evaluated using online survey forms. Data were collected using a sociodemographic information form, the UCLA Loneliness Scale, the Depression Anxiety Stress Scale, and the Difficulties in Emotion Regulation Scale. In addition to correlation analyses and group comparisons, mediation analyses were conducted using the bootstrap method.

**Results:**

Emotion regulation difficulties and loneliness were significantly positively associated with depression, anxiety, and stress. An increase in the number of days working remotely per week increased anxiety levels, and emotion regulation difficulties mediated this effect. Loneliness had a mediating role in the relationship between emotion regulation difficulties and depression.

**Conclusions:**

Emotion regulation difficulties and loneliness have a significant impact on symptoms of depression, anxiety, and stress in individuals working remotely. These findings support the need to strengthen emotion regulation skills and develop policies that increase social interaction to support the mental health of remote workers.

## Background

Remote work is a type of work in which employees perform their professional work away from the office environment, usually at home or in another location, utilizing various information and communication technologies [1]. In this work model, employees communicate with their colleagues, managers, or customers in a technological environment [2]. In addition, employees can work both from the office and remotely, i.e., in a hybrid way [3]. With the COVID-19 pandemic, the remote work model has emerged as a prominent trend in many sectors and has become a permanent fixture in business life. The remote work model has been accepted as an efficient and sustainable work method in various sectors [4–6].

The remote work model can affect employees’ work-life balance and social aspects. This model can lead to psychologically significant consequences, including social isolation, loneliness, difficulties in achieving a work-life balance, changes in daily routine, and psychological pressure [7]. Researchers have demonstrated that remote work can have a positive or negative impact on mental health. It has been demonstrated that remote work can enhance employees’ quality of life by mitigating work-related conflict and stress [8]. On the other hand, numerous studies have reported an increase in loneliness, social isolation, and psychological tension with the spread of remote work [9–11]. Remote work can lead to social and professional isolation, anxiety about professional success, decreased communication with colleagues, increased mental distress, and stress levels [12].

It has also been reported in the literature that the risk of anxiety and depression is higher in remote workers. This rate was found to be especially high in individuals living alone. Anxiety rates were found to be higher in men, and depression rates were found to be higher in women working remotely [13]. Additionally, individuals working from home have higher levels of anxiety, depression, and stress than those working in a hybrid or office setting [14]. It has been shown that high levels of anxiety, depression, and stress in remote workers may be associated with poor sleep quality, increased workload, difficulty concentrating on work, low physical activity, and workplace loneliness [15, 16].

Another psychosocial adverse effect that the remote work model can create in individuals is loneliness. Results from previous studies indicate that the spread of the remote work model has led to increased social isolation and loneliness [17, 18]. Loneliness is particularly high among individuals who work remotely four days or more per week [18]. A lack of social support at work has been cited as one of the primary reasons for loneliness in the remote work model. Support from coworkers and managers has been reported to reduce loneliness significantly [19]. Given the negative relationship between loneliness and psychological well-being [20], reduced social interaction and increased social isolation for remote workers may pose a significant risk to mental health. Loneliness among remote workers is associated with emotional exhaustion, stress, anxiety, depression, decreased job satisfaction, and poor work-life balance [21]. In employees with high levels of loneliness, commitment to work and motivation decrease. This situation also negatively affects behaviors beyond just doing their own tasks and contributing voluntarily to the organization (e.g., helping others, taking initiative, etc.) [22].

Another critical factor to consider for remote workers is emotion regulation. Emotion regulation is the capacity of individuals to monitor, evaluate, and modify their emotional responses [23]. The social isolation, disruptions in communication, and uncertainties that come with working remotely can make it difficult for individuals to regulate their emotions, paving the way for various mental health problems [24]. At the same time, individuals working remotely require more emotion regulation skills to cope with their workload, tolerate uncertainty, and compensate for deficiencies in social relationships [25].

Findings from previous studies suggest that there are significant positive relationships between emotion regulation difficulties, depressive symptoms, anxiety, and chronic stress [26, 27]. Anxiety is particularly associated with the inability to maintain a distraction strategy, while depression is associated with rumination and the persistence of negative emotions [28, 29]. Frequent use of maladaptive emotion regulation strategies (e.g., suppression, avoidance, rumination) increases symptoms of depression and anxiety. In contrast, the low use of adaptive strategies (e.g., reappraisal, problem-solving, acceptance) hinders the decrease in these symptoms [29, 30]. It has been reported that negative emotion regulation strategies, such as expressive suppression, are associated with anxiety levels in individuals receiving remote education during the COVID-19 process [31].

Emotion regulation skills have a strong and reciprocal relationship with loneliness. While some findings suggest that strong emotion regulation skills can reduce loneliness, others have reported that individuals with higher levels of loneliness use maladaptive emotion regulation strategies more frequently [30, 32]. Emotion regulation difficulties are a significant predictor of loneliness. Individuals experiencing loneliness tend to use more introverted and dysfunctional strategies to cope with negative emotions. These strategies can often perpetuate or increase loneliness rather than reduce it. For example, individuals who suppress their emotions or avoid social interactions to cope with loneliness may have more difficulty forming social connections [32]. When considered together, loneliness and emotion regulation difficulties may exacerbate anxiety, depression, and stress levels.

In light of these data, it is essential to comprehensively evaluate the relationship between loneliness and emotion regulation difficulties and depression, anxiety, and stress levels in remote workers. In particular, the increasing rate of remote work at the global level, combined with the fact that this work model has become permanent in the post-pandemic period, necessitates a more in-depth examination of these relationships in terms of employees’ mental health. Although the existing literature has examined the bilateral relationships of these psychosocial variables, studies conducted together, especially on samples of individuals working remotely, are limited. This study aims to reveal the effects of remote work on individuals’ loneliness and emotion regulation difficulties and to investigate how these two variables affect depression, anxiety, and stress levels. We will also examine the effects of some sociodemographic characteristics on these variables. Thus, the study aims to contribute to the literature on the impact of remote work on mental health, to help identify psychosocial risks early, and to identify areas of intervention to strengthen the psychosocial resources of employees.

## Methods

### Study design and data collection

This study is an analytical cross-sectional observational study. The sample consists of individuals working remotely. The sample selection was carried out using the snowball sampling method. Participants were reached through online platforms (social media, e-mail groups, etc.). A selection process based on voluntary participation was followed by providing written information about the purpose of the study. Online consent was obtained from the participants, and the data collection process was carried out through digital survey forms.

The study included 121 remote workers. The inclusion criteria were being between 18 and 65 years old, having the mental capacity to understand and respond to instructions, being willing and able to participate in the study, and working remotely for at least six months. Exclusion criteria were incomplete survey responses and working outside a remote work model.

The sociodemographic data form, the UCLA Loneliness Scale (UCLA-20), the Depression Anxiety Stress Scale (DASS-21), and the Difficulties in Emotion Regulation Scale (DERS-16) were applied to remote workers who agreed to participate in the study via online platforms.

Ethical approval was obtained from the Giresun University Training and Research Hospital Clinical Research Ethics Committee, with the number 05.03.2025/11, on March 5, 2025.

### Measures

#### Sociodemographic data form

The researchers created this form to evaluate sociodemographic variables such as gender, age, place of residence, marital status, education status, employment status, income status, lifestyle, alcohol and cigarette use, presence of physical and psychiatric disorders, number of years working remotely, and number of days working remotely per week.

#### UCLA loneliness scale

The participants’ loneliness levels were assessed with the UCLA-20, developed by Russell et al. [33] and adapted to Turkish by Demir [34]. This scale is a 20-item, 4-point Likert-type self-report instrument. Higher scores on the scale indicate that the individual experiences more intense loneliness. The internal consistency of the Turkish form of the scale was found to be high, and the Cronbach alpha coefficient was reported as 0.96.

#### Depression anxiety stress scale

The participants’ depression, anxiety, and stress levels were assessed with the DASS-21 developed by Lovibond and Lovibond [35] and adapted to Turkish by Yılmaz et al. [36]. This self-report scale consists of 21 items, graded on a 4-point Likert-type scale. The scale consists of three sub-dimensions, each with seven items: depression, anxiety, and stress. Higher scores on the scale indicate that the relevant mental health symptoms are experienced more severely. In the internal consistency analyses of the Turkish version of the DASS-21, Cronbach’s alpha coefficients for the subscales were determined as 0.87 for depression, 0.85 for anxiety, and 0.81 for stress.

#### Difficulties in emotion regulation scale

The participants’ emotion regulation difficulties were assessed using the DERS-16, developed by Gratz and Roemer [37], adapted into a short form by Bjureberg et al. [38], and translated into Turkish by Yiğit and Guzey Yiğit [39]. This self-report scale consists of 16 items. The scale, which is graded as a 5-point Likert-type scale, includes five subdimensions in its Turkish form: non-acceptance, goals, impulse, strategies, and clarity. The total DERS-16 score reflects individuals’ general emotion regulation difficulties, while the subdimensions assess specific components of these difficulties. Higher scores on the scale indicate more severe emotion regulation difficulties. In the psychometric evaluation of the Turkish form, the scale’s overall internal consistency was high, and the Cronbach alpha coefficient was reported as 0.92.

### Statistical analyses

Statistical analyses were performed using IBM SPSS Statistics 27 and PROCESS Macro. Categorical variables are shown with frequency and percentage distributions. Numerical variables are shown with mean, standard deviation, median, and interquartile range values. The conformity of the data to normal distribution was assessed with skewness and kurtosis values. If the skewness and kurtosis values ​​were between − 1.5 and + 1.5, the data were considered to be normally distributed. In pairwise comparisons, when the assumption of normality was met, the independent sample t-test was used, and when the assumption of normality was not met, the Mann-Whitney U test was used. Relationships between variables that followed normal distribution were examined with Pearson correlation analysis, and relationships between variables that did not follow normal distribution were examined with Spearman correlation analysis. In addition, mediation analyses were performed using the 5000-resampling method with the bootstrap approach at a 95% confidence interval. A two-way alpha margin of error was accepted as 0.05 in all tests.

## Results

The distribution of categorical sociodemographic and health-related variables is presented in Table 1. The sample was relatively balanced in terms of gender, and the vast majority of participants resided in urban areas. Notably, over 40% reported regular smoking.

**Table 1:** Distribution of categorical sociodemographic and health-related variables

		*n* = 121	%
Gender	Female	65	53.7
	Male	56	46.3
Place of residence	City center	120	99.2
	Village-Town	1	0.8
Marital status	Married	43	35.5
	Single	78	64.5
Lifestyle	With someone	94	77.7
	Alone	27	22.3
Physical illness	No	113	93.4
	Yes	8	6.6
Psychiatric disorder	No	106	87.6
	Yes	15	12.4
Regular smoking	No	71	58.7
	Yes	50	41.3
Regular alcohol use	No	99	81.8
	Yes	22	18.2
Regular substance use	No	118	97.5
	Yes	3	2.5
Regular exercising	No	75	62
	Yes	46	38

n: sample size

Table 2 presents descriptive statistics for continuous variables, including demographic information, remote work characteristics, and scale scores related to mental health symptoms, loneliness, and emotion regulation difficulties.

**Table 2:** Descriptive statistics of continuous sociodemographic and psychosocial variables

	Mean	Standard deviation
Age	31.20	6.02
Education (years)	16.19	2.01
Number of years working remotely	3.48	1.52
Number of days working remotely per week	4.49	1.42
Monthly income (times minimum wage)	3.77	2.18
DASS-21 Depression	7.26	4.64
DASS-21 Anxiety	5.18	4.14
DASS-21 Stress	7.69	4.44
UCLA-20 Loneliness	40.83	9.91
DERS-16 Clarity	4.32	1.70
DERS-16 Goals	8.25	3.48
DERS-16 Impulse	5.76	3.30
DERS-16 Strategies	10.49	5.10
DERS-16 Non-acceptance	6.29	3.13
DERS-16 total	35.11	14.63

*DASS-21* Depression Anxiety Stress Scale, *UCLA-20* UCLA Loneliness Scale, DERS-16: Difficulties in Emotion Regulation Scale

In pairwise comparisons, no statistically significant difference existed in DASS-21, UCLA-20, and DERS-16 variables except for regular cigarette use. Psychosocial scale scores were higher in smokers than in non-smokers; DASS-21 depression (t = −2.563, *p* = 0.012), DASS-21 anxiety (Z = −2.842, *p* = 0.004), DASS-21 stress (t = −2.361, *p* = 0.020), DERS-16 strategies (t = −2.192, *p* = 0.031), DERS-16 non-acceptance (t = −2.316, *p* = 0.023), DERS-16 impulse (t = −2.272, *p* = 0.026), DERS-16 total (t = −2.272, *p* = 0.026).

Table 3 compares whether there are significant differences in terms of mental health symptoms (depression, anxiety, stress), loneliness, and emotion regulation difficulties between individuals working remotely full-time (*n* = 73) and individuals working in a hybrid model (*n* = 48). Anxiety levels and some emotion regulation difficulties were significantly higher among full-time remote workers than hybrid workers.

**Table 3:** Comparison of full-time and hybrid employees concerning mental health symptoms, loneliness, and emotion regulation difficulties variables

	Full time (*n* = 73)	Hybrid (*n* = 48)	Statistics	*p*
	Mean ± SD/Median (Q1-Q3)			
DASS-21 Depression	7.58 ± 4.74	6.79 ± 4.91	t = 0.909	0.365
DASS-21 Anxiety	5 (3–8)	3.5 (1–6)	Z = −2.185	**0.029**
DASS-21 Stress	7.99 ± 4.34	7.23 ± 4.60	t = 0.917	0.361
UCLA-20 Loneliness	42.08 ± 10.09	38.94 ± 9.41	t = 1.722	0.088
DERS-16 Clarity	4 (3–5)	4 (3–5)	Z = −0.071	0.944
DERS-16 Goals	8.67 ± 3.58	7.60 ± 3.25	t = 1.664	0.099
DERS-16 Impulse	6.36 ± 3.61	4.85 ± 2.55	t = 2.679	**0.008**
DERS-16 Strategies	11.33 ± 5.48	9.21 ± 4.19	t = 2.278	**0.025**
DERS-16 Non-acceptance	6.67 ± 3.47	5.71 ± 2.43	t = 1.793	0.075
DERS-16 total	37.37 ± 15.66	31.67 ± 12.27	t = 2.129	**0.035**

*DASS-21* Depression Anxiety Stress Scale, *UCLA-20* UCLA Loneliness Scale, *DERS-16* Difficulties in Emotion Regulation Scale

*SD* Standard deviation, *Q1* First quartile, *Q3* Third quartile

Table 4 presents the correlation coefficients between emotion regulation difficulties, loneliness, mental health symptoms, and the number of days working remotely. Significant positive correlations were found between DERS-16 scores and all subscales of the DASS-21. Similarly, loneliness scores showed significant positive correlations with mental health symptoms and emotion regulation difficulties. Notably, the number of days working remotely per week was positively correlated with anxiety and the impulse subdimension of the DERS-16.

**Table 4:** Correlation coefficients of numerical variables in the sample

	DERS-16 total	DERS-16 Non-acceptance	DERS-16 Strategies	DERS-16 Impulse	DERS-16 Goals	DERS-16 Clarity	UCLA-20 Loneliness	DASS-21 Stress	DASS-21 Anxiety
Number of days working remotely per week	0.222*	0.193*	0.219*	0.301*					0.319**
DASS-21 Depression	0.662**	0.413**	0.680**	0.490**	0.489**	0.451**	0.557**	0.713**	0.561**
DASS-21 Anxiety	0.565**	0.439**	0.557**	0.522**	0.438**	0.409**	0.343**	0.752**	
DASS-21 Stress	0.618**	0.459**	0.610**	0.549**	0.521**	0.435**	0.428**		
UCLA-20 Loneliness	0.509**	0.331**	0.567**	0.464**	0.380**	0.373**			

*DASS-21* Depression Anxiety Stress Scale, *UCLA-20* UCLA Loneliness Scale, *DERS-16* Difficulties in Emotion Regulation Scale

* *p* < 0.05

** *p* < 0.01

**Fig. 1:**
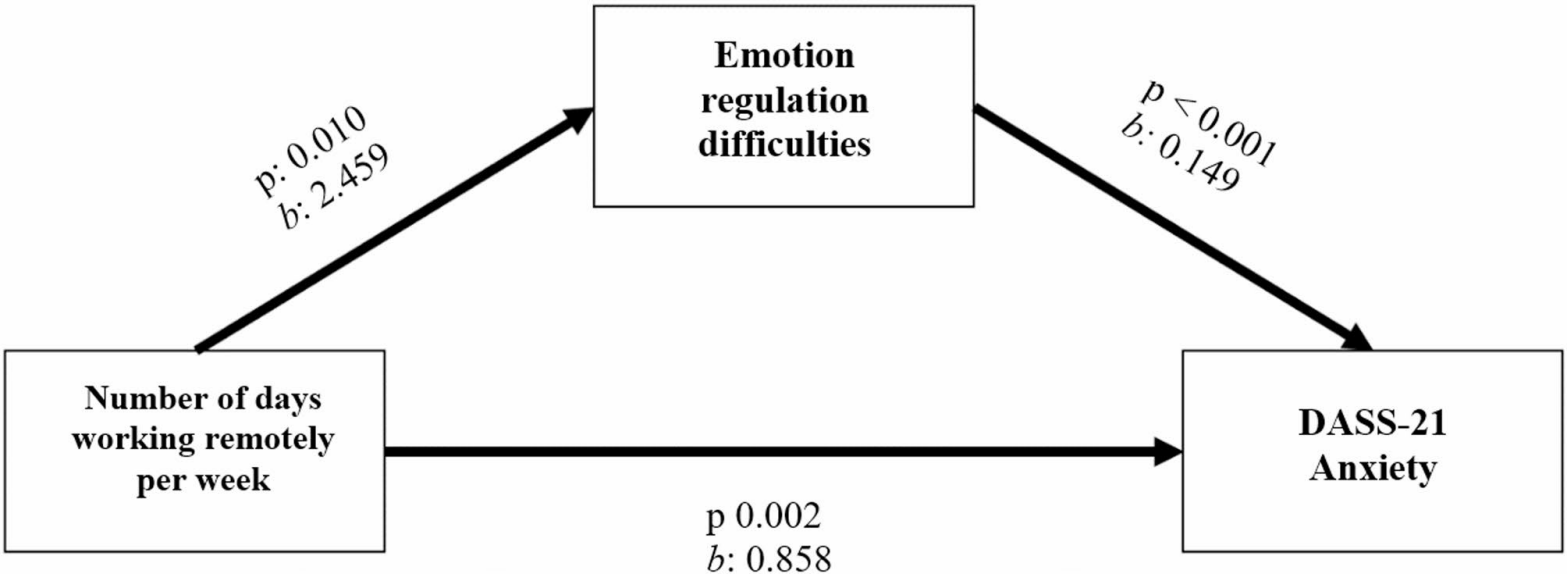
Mediation model of the relationship between the number of days working remotely and anxiety. Direct effect: *b*: 0.492, p: 0.035. Indirect effect: *b*: 0.366, 95% CI: 0.087–0.713. *b*: Unstandardized beta coefficient, p: Significance level

The model illustrating the mediating effect of emotion regulation difficulties on the relationship between the number of days working remotely per week and anxiety level is visualized in Fig. 1. The model was statistically significant (*p* < 0.001) and explained approximately 35% of the effect on anxiety level (R² = 0.345). The total effect (*b* = 0.858, *p* = 0.002) and direct effect (*b* = 0.492, *p* = 0.035) of the number of days working remotely per week on the anxiety level were statistically significant. According to the model, a one-unit increase in the number of days working remotely per week directly causes a 0.492-unit increase in anxiety level. In addition, the indirect effect of the number of days working remotely per week on anxiety level, mediated by emotion regulation difficulties, was found to be statistically significant (*b* = 0.366, 95% CI = 0.087–0.713). This mediation effect remained significant when the smoking variable was statistically controlled (95% CI = 0.062–0.651). As a result, it was determined that the number of days working remotely per week had a mediating role in the effect of emotion regulation difficulties on anxiety levels. The full standardized effect size value (K²) of this mediating effect was 0.124, which can be said to indicate a medium-level effect.

**Fig. 2:**
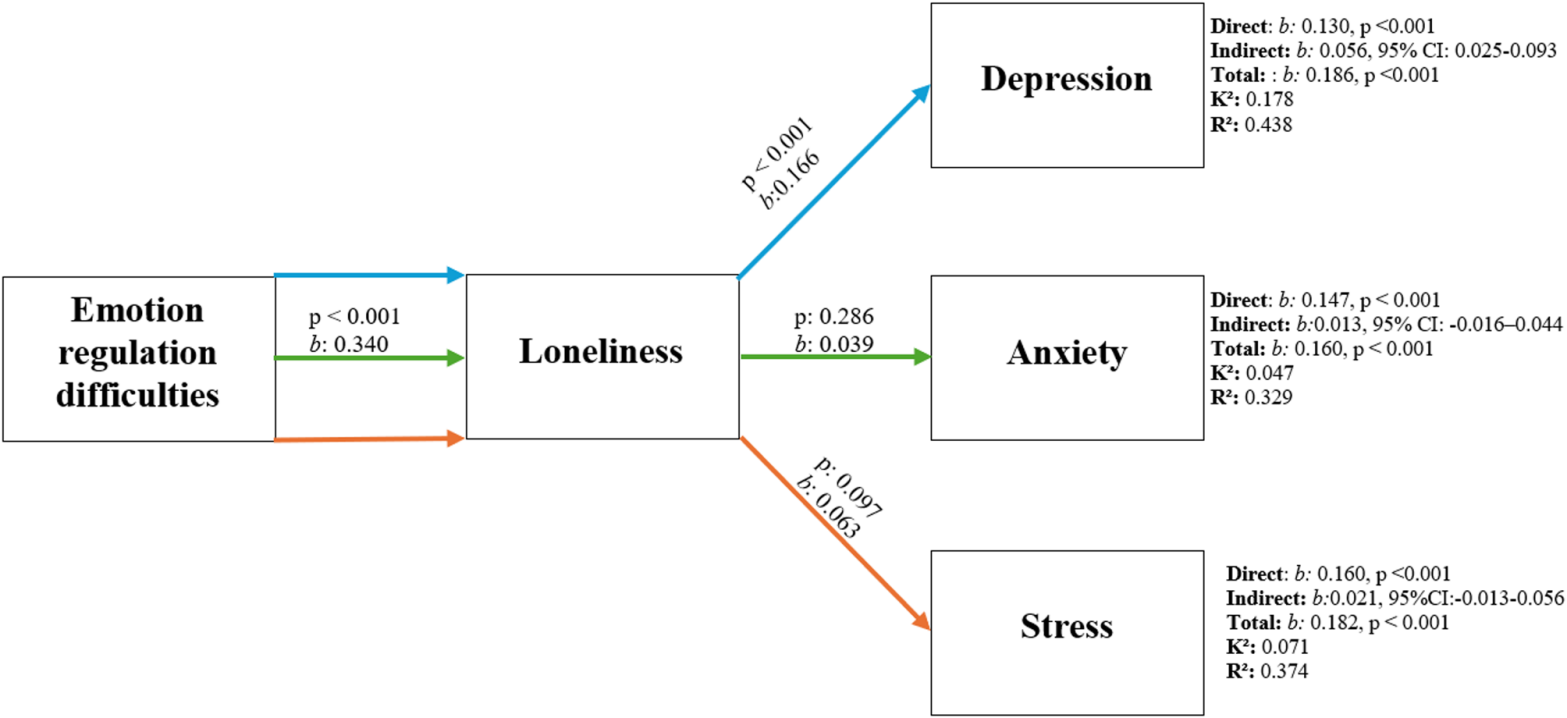
Mediation model of the relationship between emotion regulation difficulties and DASS-21 dimensions. *b*: Unstandardized beta coefficient, p: Significance level

The models created to show the mediating effect of loneliness level on the relationship between emotion regulation difficulties and depression, anxiety, and stress levels are visualized in Fig. 2. The models are statistically significant (*p* < 0.001). According to the mediation analysis results, no mediating role of loneliness in the relationship between emotion regulation difficulties and anxiety and stress levels was determined (95% CI −0.016–0.044; −0.013-0.056, respectively). The total effect of emotion regulation difficulties on depression level (*b* = 0.186, *p* < 0.001), its direct effect (*b* = 0.130, *p* < 0.001), and its indirect effect through loneliness level (*b* = 0.056, 95% CI = 0.025–0.093) were found to be statistically significant. This mediation effect remained significant when the smoking variable was statistically controlled (95% CI = 0.026–0.093). In other words, loneliness was found to mediate the relationship between emotion regulation difficulties and depression.

## Discussion

This study examined the effects of emotion regulation difficulties and loneliness on depression, anxiety, and stress levels in remote workers. The findings show that emotion regulation difficulties in remote workers are closely related to higher levels of depression, anxiety, and stress, independent of loneliness. Loneliness also exhibited positive relationships with all DASS-21 subscales and had a significant mediating effect on the relationship between emotion regulation difficulties and depression. Additionally, increased weekly remote work hours were found to be a risk factor for increased anxiety levels, with some of this effect occurring through emotion regulation difficulties. In addition, some lifestyle factors, such as regular smoking, were found to be associated with emotion regulation difficulties and higher levels of depression, anxiety, and stress.

The relationship we found between emotion regulation difficulties and mental health symptom levels has been frequently identified in the literature before. It has been stated that emotion regulation difficulties function as a transdiagnostic phenomenon in the context of mental health symptoms [30, 40]. Negative emotion regulation strategies that are not solution-focused may contribute to the intensification of negative emotions and lead to the development of maladaptive coping strategies. As a result, symptoms of anxiety and depression may become more severe. Although the relationship between emotion regulation difficulties and mental health symptoms is well known, studies conducted with individuals working remotely in this context have found little space in the literature. Increased levels of depression, anxiety, and stress are linked to emotion regulation difficulties, suggesting that individuals who work from home for many days experience additional psychological strain when having to cope with workload and uncertainty alone. These findings indicate that personal coping skills may shape the relationship between remote work and mental health.

Our finding that symptoms of depression, anxiety, and stress increase when loneliness increases is consistent with previous studies demonstrating the adverse effects of workplace loneliness on mental health in remote workers [15]. Loneliness in remote work is shaped not only by physical isolation but also by multi-layered mechanisms such as energy depletion, role conflicts, and reduced social relationships [17]. In addition, there is a significant relationship between loneliness and emotion regulation difficulties. Individuals experiencing loneliness often use more maladaptive emotion regulation strategies. One study reported that emotion regulation difficulties explained approximately half of the variance in loneliness [32]. As with other study results, our findings revealed a positive correlation between emotion regulation difficulties and loneliness among remote workers. One factor affecting the mental health of remote workers is the number of days they work remotely. As the number of days they work remotely increases, their mental well-being may decrease [18, 41]. However, studies examining the relationship between remote work time and depression, anxiety, and stress are limited. We found that the number of days working remotely was positively correlated with anxiety level and emotion regulation difficulties sub-dimensions, such as impulse, strategy, and non-acceptance. Considering that remote work may be a risk factor for the deterioration of mental well-being, it seems logical that the increase in remote work time is correlated with the increase in anxiety levels. Individual characteristics or emotion regulation difficulties that may arise from working remotely, such as difficulty controlling impulsive behaviors, feeling unable to achieve effective strategies to manage negative emotions, denying the negative emotions they experience, or blaming themselves for these emotions, may explain the increase in anxiety levels. Additionally, remote workers may lack these skills because they implement emotion regulation strategies through a digital environment [42]. In line with these findings, we found that anxiety levels and emotion regulation difficulties scores were lower in individuals working in the hybrid model compared to individuals working full-time remotely. Our findings suggest that part of this anxiety-related impact may be mediated by the emotion regulation difficulties inherent in remote work. That is, increased anxiety levels in those who work from home for large numbers of days appear to be related in part to the difficulties these individuals have in managing their emotions. To our knowledge, this is a novel finding in the literature, as previous studies have not explained the relationship between the number of days working remotely and anxiety through emotion regulation difficulties. However, considering the direct effect of the number of days working remotely on the anxiety level, it can be said that emotion regulation difficulties cannot entirely explain this relationship. Other factors related to this relationship should be examined in further studies.

Depression, anxiety, stress, and emotion regulation difficulties scores were significantly higher in individuals with smoking habits. Smoking is common among remote workers. This condition is associated with easier access to smoking, reduced social support, and dysfunctional coping strategies [43]. There is a bidirectional positive relationship between mental health symptoms and smoking in terms of biological and psychosocial mechanisms [44]. In addition, smoking is closely related to emotion regulation difficulties [45]. This may be related to the fact that stressed and anxious individuals often prefer smoking as a coping mechanism. However, more research is needed before a causal interpretation can be made, especially regarding remote workers.

The relationships between loneliness, emotion regulation difficulties, and symptoms such as depression and anxiety are complex. In the mediation model we created to clarify this complexity, we found that loneliness has a mediating role in the effect of emotion regulation difficulties on depression. In other words, some of the depressive symptoms that increase with emotion regulation difficulties can be explained by the loneliness that individuals experience. According to the model we created, emotion regulation difficulties may cause loneliness, and loneliness may cause depressive symptoms. As loneliness increases, people may have more difficulty coping with their negative emotions and develop depressive symptoms. However, this finding should be interpreted cautiously due to the study’s cross-sectional design. Nevertheless, this result provides an original contribution to the literature. This mediating relationship offers a holistic perspective on how emotional and social factors can interact to impact mental health in a remote work environment. In addition, the findings show that emotion regulation difficulties are closely related to high levels of depression, anxiety, and stress, regardless of the loneliness. In other words, remote workers experiencing emotion regulation difficulties reported higher levels of depressive symptoms, anxiety, and stress, even if they did not experience loneliness. This finding highlights the importance of developing and enhancing emotion regulation skills for individuals working remotely.

The relationships between emotion regulation difficulties and gender [46], lifestyle [47], and exercise [48] have been previously reported. Exercise has been reported to be associated with loneliness [49]. In addition, women have been found to experience greater loneliness as a result of social isolation [50], and those without a romantic relationship or living alone also experience greater loneliness [33, 51]. However, we did not find any significant effects of social variables such as gender, marital status, lifestyle, and exercise on emotion regulation difficulties and loneliness. Studies on these variables in individuals working remotely are limited in the literature, and our results may represent a different group from the general population. Contrary to existing literature, we did not find any significant effects of gender, marital status, lifestyle, and exercise on depression, anxiety, or stress levels in remote workers [13, 15, 52]. Similarly, we found no significant relationship between remote work and loneliness or between full-time work and depression, stress, and loneliness. Additionally, loneliness did not mediate the relationship between emotion regulation difficulties and anxiety and stress. These contradictory findings suggest that the effects of remote work may vary depending on context and individual circumstances. Many factors not assessed in our study (e.g., social support, working conditions, self-discipline) may modify the effects of remote work on mental health. Therefore, the discrepancy between the results reported in the literature and our findings suggests that the nature of the remote work experience and the employees’ personal/organizational resources may vary. The small sample size may have prevented some results from reaching significance. Additionally, the fact that our study focused more on risk factors and the sample characteristics (mostly full-time remote workers) may have highlighted the adverse effects of remote work more clearly.

The findings offer some theoretical and practical implications. In the context of remote work, the role of emotion regulation and the need for social togetherness in mental health is confirmed in line with the literature [53, 54]. Attachment and social needs theories predict that when individuals’ needs for belonging and support are unmet (for example, when they experience loneliness), their psychological well-being will be damaged [55]. Additionally, individuals experience psychological distress when their stress-coping and emotion-regulation skills are inadequate [56]. The findings indicate that individuals who face social isolation in a remote work environment are more likely to have mental health problems if their emotion regulation skills are weak. This result highlights that, in addition to the freedom and flexibility that remote work brings, employees’ needs for building relationships and managing emotions are equally important. From a practical perspective, our findings suggest the need for targeted interventions at both individual and organizational levels to protect mental health in remote work environments. In particular, supporting employees’ emotion regulation skills and increasing their opportunities for social interaction can play a critical role in reducing levels of depression, anxiety, and stress. The physical separation of remote workers can often lead to emotional and social needs not being fully met despite the use of communication technologies. Therefore, it is essential to provide training and resources that enable employees to manage their emotions in a healthy manner, as well as to develop practices that reinforce a sense of belonging in the workplace. As remote work becomes more widespread, employers should develop supportive policies that address the psychosocial needs of employees. Mental health professionals can support employees’ mental well-being by offering specific intervention programs that address loneliness and emotion regulation difficulties. Policymakers should create regulations that encourage employers to protect the mental health of their employees and raise public awareness through awareness campaigns. Thus, the mental health of individuals can be protected more effectively in remote work conditions. In conclusion, the interactions revealed by our research demonstrate that the development and strengthening of emotional skills, as well as social ties, are essential for enhancing psychological resilience among remote workers.

### Limitations and future directions

This study has several limitations. First, the cross-sectional design prevents any conclusions about causality. Second, the reliance on self-report measures may have introduced bias due to social desirability or common method variance. Third, the sample was relatively small and demographically homogeneous (e.g., predominantly urban and educated), which may limit the generalizability of the findings. Additionally, considering that some participants transitioned to a hybrid model or had varying lengths of remote work experience, it became challenging to determine the impact of variables such as the number of years of remote work experience. Lastly, objective assessments such as clinical interviews were not included. Future studies should address these limitations with longitudinal designs, larger and more diverse samples, and multimethod approaches.

## Conclusions

In today’s world, where remote work is becoming increasingly widespread, developing strategies to protect the mental well-being of employees has become critical. Our findings provide an important framework for understanding the relationship between emotion regulation difficulties, loneliness, and mental health during remote work. Based on our results, we recommend (1) supporting employees in developing emotion regulation skills through training and resources, (2) implementing organizational policies that promote social interaction and reduce workplace isolation, and (3) encouraging mental health professionals to develop targeted interventions for remote workers. Further longitudinal research is needed to understand better the directionality and long-term consequences of these psychosocial dynamics.

## Data Availability

The datasets used during the current study are available from the corresponding author on reasonable request.

## Abbreviations

DASS-21: Depression Anxiety Stress Scale
UCLA-20: UCLA Loneliness Scale
DERS-16: Difficulties in Emotion Regulation Scale
SPSS: Statistical Package for the Social Sciences
CI: Confidence interval
SD: Standard deviation
Q1/Q3: First quartile / third quartile
b: Unstandardized beta coefficient
R²: Coefficient of determination (explained variance)
K²: Standardized effect size in mediation analysis
p: p-value (statistical significance level)
Z: Z-score (used in non-parametric tests)
t: t-value (used in t-tests)
